# Cold Air Pre-Cooling Extends Postharvest Shelf Life of *Volvariella volvacea* by Maintaining Energy Metabolism Homeostasis

**DOI:** 10.3390/foods15061077

**Published:** 2026-03-19

**Authors:** Wubo Yang, Yuanyuan Li, Wenhan Wang, Jingsong Zhang, Ming Gong, Wei Jia

**Affiliations:** 1 Institute of Edible Fungi, Shanghai Academy of Agricultural Sciences, Shanghai 201403, China; yaaabu@163.com (W.Y.); wangwenhan@saas.sh.cn (W.W.); syja16@saas.sh.cn (J.Z.); 2 College of Food Science and Technology, Shanghai Ocean University, Shanghai 201306, China; liyy0323@163.com

**Keywords:** *V. volvacea*, cold-air pre-cooling, mitochondria, energy metabolism

## Abstract

This study investigated the preservative effect and molecular mechanism of cold-air pre-cooling (CAP) combined with storage at 15 °C/85% relative humidity on *Volvariella volvacea*. CAP significantly reduced weight loss and browning, maintained moderate respiratory intensity, minimised malondialdehyde accumulation and polyphenol oxidase activity, and preserved higher firmness and soluble-protein content, extending the shelf life by 4 d. An analysis of energy metabolism indices revealed that CAP increased mitochondrial quantity, membrane potential, and ATP content. Specifically, CAP restricted the tricarboxylic acid (TCA) cycle rate by downregulating the activities of succinate dehydrogenase, isocitrate dehydrogenase, and citrate synthase. Additionally, CAP prevented the peak activation of respiratory complex I, while sustaining optimal activity of complexes III and IV, thereby stabilising intracellular ATP levels. Transcriptomic analysis further indicated that CAP suppressed the activity of the TCA cycle and oxidative phosphorylation pathways during postharvest storage. Quantitative real-time PCR (qPCR) validated the downregulation of genes associated with respiratory complexes after CAP treatment. In conclusion, CAP maintained the postharvest quality of *V. volvacea* by preserving energy metabolism homeostasis, providing a theoretical basis for its application in edible mushroom preservation.

## 1. Introduction

After harvest, metabolic activity continues within the fruiting bodies of edible fungi, and their respiratory rate remains remarkably high [1]. As the primary site of aerobic respiration in cells, mitochondria play a pivotal role in cellular energy metabolism. Post harvest, respiration is the only energy-supplying pathway for edible fungi. It not only provides the energy essential for maintaining cellular life activities such as substance transport and cellular repair but also greatly contributes to the synthesis of various organic compounds, supplying key precursors and reducing agents. However, during respiration, substantial heat is released, which can elevate the temperature, accelerate metabolic rates, enhance the activity of certain enzymes, and potentially lead to more rapid quality deterioration, thereby shortening the shelf life of edible fungi [2]. Therefore, respiration intensity plays a decisive role in determining the quality of edible fungi. Typically, maintaining quality involves reducing the respiratory rate. The respiratory metabolic pathways in edible fungi include glycolysis, the tricarboxylic acid (TCA) cycle, and the respiratory chain.

*Volvariella volvacea*, a highly prized edible mushroom, is known for its exceptional flavour, intense aroma, and rich nutritional profile, underscoring its potential for both culinary and medicinal applications [3]. As a thermophilic species, it exhibits rapid softening and liquefaction under conventional refrigeration conditions (0–4 °C) owing to cryogenic autolysis. Conversely, elevated storage temperatures promote continued cap expansion, leading to membrane rupture and premature opening of fruiting bodies [4]. These phenomena result in a marked decline in texture, flavour, and overall quality, ultimately diminishing the economic value. The dual challenges of cryogenic autolysis and high-temperature premature opening significantly hinder postharvest storage and transportation, thereby constraining the industry’s development.

To mitigate postharvest quality deterioration in *V. volvacea*, studies have explored various preservation techniques, including alternating magnetic fields [5], static-magnetic-field treatment [6], starch-based composite films [7], and irradiation [8]. However, because of food safety and environmental sustainability considerations, the most widely adopted preservation method for *V. volvacea* is storage at 15 °C. This temperature effectively reduces the respiratory rate of mushrooms while extending postharvest shelf life [9].

Pre-cooling is the rapid removal of field heat from fruits and vegetables shortly after harvest, typically by exposing them to cold air at 0–5 °C for 2–24 h, with the goal of quickly lowering the product’s core temperature to the target storage level. This process helps suppress respiratory activity, reduce water loss, and delay ripening, thereby extending shelf life. The exact temperature and duration depend on the commodity, but the treatment is considered complete when the centre temperature reaches within ±1 °C of the desired storage temperature [9]. The exact temperature and duration depend on the commodity, but the treatment is considered complete when the center temperature reaches within ±1 °C of the desired storage temperature. Chen et al. [10] demonstrated that pre-cooling effectively reduced the decay index of Chinese bayberry fruits postharvest, inhibited the respiratory rate and ethylene release, and delayed ripening, thereby extending the shelf life. Wang et al. [11] found that pre-cooling reduced the respiratory intensity of *Tricholoma matsutake*. He et al. [12] reported that pre-cooling lowered the activity of polyphenol oxidase (PPO) during the storage of *Agaricus bisporus*. Khan et al. [13] found that pre-cooling reduced the weight loss and opening rate of paddy *V. volvacea* postharvest. Cold-air pre-cooling (CAP) is currently the most common employed pre-cooling method. It has the advantages of simple operation, high efficiency, and strong adaptability, and it is widely used for fruit and vegetable preservation. Phoengmak et al. [14] studied the temperature effect of CAP on the preservation of postharvest durian and found that the optimal preservation temperature was 1 °C.

While conventional mushroom preservation primarily relies on cold storage and modified atmosphere packaging [15], enhancing postharvest preservation of edible mushrooms is critical to extending their freshness by slowing metabolic processes in fruiting bodies [16]. Previous studies have demonstrated that low temperatures reduce glycolytic enzyme activity, modulate glycolytic flux, and redirect carbon metabolism toward the synthesis of well-characterized stress-resistant compounds such as trehalose in *Paxillus involutus* and *V. volvacea* [17]. As the foundational stage of postharvest thermal regulation, pre-cooling acts as a critical defense to retard metabolic processes and maintain produce quality. However, the dynamic metabolic responses triggered by pre-cooling as a transient treatment remain largely uncharacterized. While standard 0–4 °C protocols suit most produce, they induce chilling injury in thermophilic *V. volvacea*. Therefore, a species-specific moderate pre-cooling strategy at 15 °C is utilized to mitigate stress.

This study investigates how optimal pre-cooling maintains energy homeostasis in *V. volvacea*, with three objectives: (1) evaluate cold air pre-cooling (CAP) combined with 15 °C/85% RH storage on postharvest quality; (2) decipher molecular mechanisms underlying energy metabolism preservation; and (3) establish a CAP-based framework for *V. volvacea* and other climacteric fungi. By bridging empirical practice and physiological understanding, this research advances targeted preservation strategies for thermophilic mushrooms.

## 2. Materials and Methods

### 2.1. Experimental Design and Sample Preparation

*V*. *volvacea* was cultivated at the Zhuanghang Integrated Experimental Station, Shanghai Academy of Agricultural Sciences, under controlled conditions of 30 ± 2 °C and 95 ± 5% relative humidity. Fruiting bodies at the egg stage were harvested after a 4-week cultivation period and transported to the laboratory within 1 h post-harvest.

For packaging, food-grade polypropylene (PP) trays ((Guangzhou Jiexin Biotechnology Co., Ltd., Guangzhou, China), dimensions: 180 mm × 130 mm × 55 mm) were used. Each tray was equipped with 40 uniformly distributed ventilation holes (3 mm in diameter), arranged in a grid pattern of 8 rows × 5 holes per row. A standardized weight of 250 g of *V. volvacea* fruiting bodies was loaded into each tray, ensuring the mushrooms were stacked to a maximum depth of two-thirds of the tray height to prevent compression damage. Subsequently, each tray was sealed using a 0.06 mm thick food-grade polyethylene (PE) film (Hangzhou Miuge Chemical Commodities Science & Technology Co., Ltd., Hangzhou, China) via heat-sealing at 120 °C for 2 s.

An experimental unit consisting of 1 kg of *V. volvacea* fruiting bodies was allocated to each container per replicate. Due to natural variation in mushroom size, the quantity was standardized by weight rather than individual count to ensure consistency across all trials. The experimental groups were set as follows:

Room-temperature (RT) treatment: Stored at 25 ± 0.5 °C and 45 ± 3% relative humidity.

Cold-air Pre-cooling (CAP) treatment: Pre-cooling with cold air to 15 ± 1 °C, followed by storage at 15 ± 0.5 °C and 85 ± 3% relative humidity.

Non-pre-cooling (NP) treatment: Stored at 15 ± 0.5 °C, 85 ± 3% relative humidity.

### 2.2. CAP Treatment

*V*. *volvacea* fruit bodies were pre-cooled using a Gree KS-10X61D fan (GREE Electric Appliances Co., Ltd., Zhuhai, China) under a controlled relative humidity of 90 ± 5%. An ice-salt mixture, serving as a cold source, was placed beneath the mushroom trays, covering no less than 80% of the area and maintaining a distance of ≤1 cm from the fruit bodies. The 30 min pre-cooling process comprised three sequential stages: (1) Intensive Cooling (10 min): 3.0 m/s airflow lowered the temperature from 30 °C to 22 °C; (2) Equilibration (10 min): 1.8 m/s airflow combined with the cold source reduced the temperature to 17 °C; and (3) Final Cooling (10 min): 1.0 m/s airflow stabilized the temperature at the target of 15 °C.

Baseline samples, designated as Day 0 (D0), were collected within 1 h post-harvest prior to the application of any treatment. For the room-temperature control (RT) group, sampling was performed only at Day 2 (48 h), corresponding to sample identifier R2, due to rapid quality deterioration at ambient conditions. For both the cold-air pre-cooling (CAP) and non-pre-cooling (NP) groups, samples were taken at Day 2 (48 h) and Day 4 (96 h) of storage, designated as Y2, Y4, N2, and N4, respectively. All procedures were conducted under aseptic conditions to minimize microbial contamination during postharvest handling.

### 2.3. Physiological Index Determination in V. volvacea

The weight loss rate of *V. volvacea* was determined using gravimetric analysis. Before sample placement, the tare weight of the container was recorded. Mushroom samples were placed in a container, and the total mass was measured at pre-storage (W) and postharvest storage (W_1_) time points, with all values recorded in kilograms (kg). The weight loss rate was calculated as follows:

(1) Weight loss rate (%) = (W − W_1_)/W × 100

The firmness of *V. volvacea* was measured using a puncture test, following a methodology described by Mohd et al. [18] using a TA. XT2i Texture Analyzer (Stable Micro Systems Ltd., Godalming, UK) at 25 °C. Five randomly selected samples from each treatment group were tested. Six equidistant points on the cap surface of each sample were punctured, and the mean value of the six measurements was recorded as the representative firmness value for that sample.

The Browning Index (BI) of *V. volvacea* was determined by measuring colour parameters at three uniformly selected points along the longitudinal section of the stipe, using a CS-250 colorimeter (Hangzhou Colour Spectrum Technology Co., Ltd., Hangzhou, China), following a methodology described by Minh et al. [5]. Seven randomly selected samples were used for each treatment group.

The respiration rate of *V. volvacea* fruiting bodies was determined using a JFQ-3150H fruit and vegetable respirometer (JunFan Instruments Co., Ltd., Beijing, China), which features non-dispersion infrared CO_2_ detection (0–2000 ppm, linearity ≤ ±2% full scale), two interchangeable chambers (Ø100 × H140 mm, Ø150 × H250 mm) for flexible sample adaptation, and real-time monitoring of CO_2_, O_2_, temperature, and humidity to support postharvest physiological studies, according to a method described by Iqbal et al. [19] with slight modifications. Fresh mushroom samples were placed in a sealed approx. 1.1 L respiration chamber (Ø100 × H140 mm) and incubated at 25 °C for 20 min. The change in CO_2_ concentration within the chamber was then measured using the JFQ-3150H fruit and vegetable respirometer, and the respiration rate was expressed as the amount of CO_2_ released per unit time per unit weight of tissue.

Soluble protein content was determined according to the manufacturer’s instructions, using a BCA Protein Concentration Assay Kit (CAS. No. 97-65-4; Cat. No. P0012-500, Beyotime Biotechnology, Shanghai, China). Three samples per group were selected, and the MDA content was determined using the thiobarbituric acid method [20]. Similarly, three samples per group were selected, and PPO activity was assayed using the catechol colorimetric method [21].

### 2.4. Energy Metabolism Index Analysis in V. volvacea

Mitochondria were isolated according to the manufacturer’s instructions, using an Upgraded Mitochondria Isolation Kit (for Tissues and Cells) (Cat. No. C0010-100, Beijing Applygen Gene Technology Co., Ltd., Beijing, China).

Mitochondria were stained using Janus Green B Staining Solution (CAS No. 2869-83-2; Cat. No. S19083-1g, ≥65% purity, Shanghai Yuanye Bio-Technology Co., Ltd., Shanghai, China), using a method by Harris [22], and observed under a light microscope. In addition, five random fields were selected for counting.

Mitochondrial ultrastructure was analyzed as follows. Approximately 1 mm^3^ of tissue from the stipe of *V. volvacea* was collected and fixed in a 2.5% glutaraldehyde solution for 6 h and then stored in a refrigerator at 4 °C. Methods described by Arregui [23] were used for section preparation and staining.

Mitochondrial membrane potential (MMP) in *V. volvacea* was measured using an MMP Assay Kit (JC-1) (Catalogue No. M8650-100t; Beijing Solarbio Science & Technology Co., Ltd., Beijing, China).

The activities of three key enzymes in the TCA cycle, namely succinate dehydrogenase (SDH), mitochondrial isocitrate dehydrogenase (ICDH), and citrate synthase (CS), were measured using commercially available assay kits (Catalogue No. BC0955-100T/96S, BC2160-50T/24S, and BC1060-100T/48S, respectively; Beijing Solarbio Science & Technology Co., Ltd., Beijing, China).

The activities of mitochondrial respiratory chain complexes I, III, and IV were measured using the commercially available Mitochondrial Respiratory Chain Complex I, III, and IV Activity Detection Kits (Microplate Method) (Catalogue No. BC0515-100T/96S, BC3245-100T/48S, and BC0940-100T/96S, respectively; Beijing Solarbio Science & Technology Co., Ltd., Beijing, China).

ATP content was measured in the fruiting bodies of *V. volvacea*, using an ATP assay kit (Microplate Method) (Catalogue No. BC0305-100T/96S; Beijing Solarbio Science & Technology Co., Ltd., Beijing, China).

### 2.5. Transcriptome Analysis

Three sample sets were re-sequenced by Shanghai OE Biotech Co., Ltd. (Shanghai, China) on an Illumina NovaSeq platform (San Diego, CA, USA). Total RNA extraction was performed as described in our previous publication [24]. Clean reads were obtained by removing adapter sequences and low-quality reads (average quality score < Q20). Reference-based transcriptomic analysis was conducted using the *V. volvacea* reference genome downloaded from the Joint Genome Institute (JGI; https://genome.jgi.doe.gov/portal/Volvo1/download/Volvo1_AssemblyScaffolds.fasta.gz, accessed on 25 September 2025). Raw gene expression counts were quantified using HTSeq v0.6.1, normalised to fragments per kilobase of transcript per million mapped reads (FPKM), and subjected to principal component analysis (PCA) using R v3.2.0. Differential gene expression was analysed using DESeq2 (version 1.38.3), and significantly differentially expressed genes (DEGs) were defined as those with q-values < 0.05 and fold changes > 2 across comparison groups. Transcriptomic analysis was performed using the OECloud platform (Shanghai OE Biotech, Shanghai, China), while Gene Set Enrichment Analysis (GSEA) was conducted using the GSEA software package (version 4.3.2) [25].

### 2.6. cDNA Synthesis for qPCR Analysis

Total RNA was reverse-transcribed into cDNA using a standard protocol. Synthesised cDNA was stored at −20 °C until subsequent quantitative real-time polymerase chain reaction (qPCR) analysis. Primers for target genes were designed based on qPCR principles and transcriptomic data (Table 1), synthesised by Shanghai Sangon Biotech (Shanghai, China). qPCR reactions (10 μL total volume) contained 5 μL 2× SYBR Green Master Mix, 0.5 μL cDNA, 0.4 μL of each forward/reverse primer (10 μM), and 3.7 μL ddH_2_O. Cycling conditions: 50 °C for 2 min, 95 °C for 10 min, followed by 40 cycles at 95 °C for 15 s and 60 °C for 1 min, with a melt curve stage (95 °C for 15 s, 60 °C for 1 min, and a ramp up to 95 °C). cDNA synthesized from the experimental groups was used as the amplification template, with glyceraldehyde-3-phosphate dehydrogenase (GAPDH) employed as the internal reference gene, consistent with a previously validated protocol [26]. The relative transcript levels of TCA cycle and mitochondrial respiratory chain genes were analysed using the 2^−ΔΔCt^ method, with three biological replicates per sample and averaged Ct values.

### 2.7. Data Processing and Statistical Analysis

Data were processed using Microsoft Excel 2010. Statistical analysis was performed using a one-way ANOVA with Tukey’s post hoc test (IBM SPSS Statistics 26, *p* < 0.05). Quantitative data were presented as mean ± SD (n = 3), with each measurement replicated thrice to ensure reliability.

## 3. Results and Discussion

### 3.1. Determination of Appearance and Physiological Indices of V. volvacea Across Different Storage Periods

**Figure 1:**
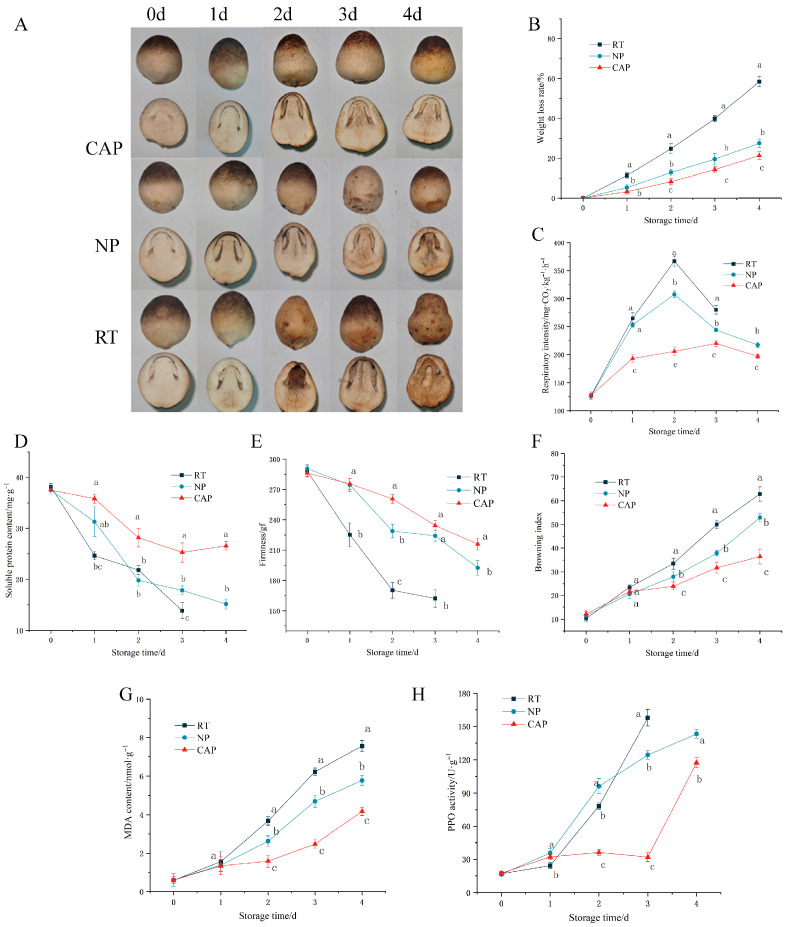
Effect of different treatments on the appearance (**A**), weight loss rate (**B**), respiratory intensity (**C**), soluble-protein content (**D**), firmness (**E**), browning degree (**F**), malondialdehyde (MDA) (**G**) and polyphenol oxidase (PPO) activity (**H**) of *V. volvacea* during different storage periods. Different letters indicate statistically significant differeneces (*p* < 0.05).

Figure 1A illustrates the comparative postharvest quality deterioration of *V. volvacea* across three storage groups: RT, NP, and CAP. On the first day of storage, RT exhibited surface desiccation and significant water loss. By the second day, RT displayed a greyish exterior, pronounced internal browning, and a soil-like odour, whereas NP showed slight longitudinal browning and CAP remained visually unchanged. On the third day, RT underwent extensive browning, emitted a mould-like odour, and developed dry, brittle surfaces, rendering it commercially nonviable. Contrastingly, NP exhibited marked internal browning and near-complete loss of aroma, while CAP maintained slight browning. By the fourth day, NP demonstrated extensive internal browning, tissue softening, and a pungent rotten odour, whereas CAP sustained partial internal browning with no exterior changes. Throughout the subsequent storage stages, the pre-cooling group consistently outperformed the other two groups in terms of appearance quality, maintaining higher surface integrity and colour stability.

*V. volvacea* is highly perishable because of its high water content, which leads to rapid postharvest water loss, peel wrinkling, and weight loss, significantly affecting its marketability. The weight loss rates of *V. volvacea* in all three groups exhibited an upward trend during storage, indicating continuous postharvest respiratory water loss (Figure 1B). RT demonstrated a significantly higher weight loss rate of 24.78% by the second day of storage, whereas the other two groups maintained relatively low weight loss rates in the early stages (12.84% for NP and 8.28% for CAP on day 2), both significantly lower than that of RT. These findings suggest that storage at 15 °C effectively delayed postharvest water loss in *V. volvacea.* Additionally, the weight loss rate of CAP was significantly lower than that of NP, with CAP maintaining a relatively low rate of 21.38% by the fourth day of storage. The results indicate that CAP can further inhibit water loss in *V. volvacea* under 15 °C storage conditions, thereby extending its shelf life. The respiratory intensity of *V. volvacea* in all three groups increased during storage, which is consistent with the characteristics of climacteric edible fungi (Figure 1C). RT exhibited the highest respiratory intensity throughout the storage period, peaking on day 2 at 366.84 mg·kg^−1^·h^−1^. NP showed a similar trend to RT, with a respiratory peak also occurring on day 2, albeit at a lower magnitude of 307.54 mg·kg^−1^·h^−1^. This indicates that lowering the storage temperature can effectively reduce respiratory activity, which aligns with findings obtained by Wang et al. [27]. In contrast, CAP displayed a relatively stable respiratory pattern with no pronounced peak, and its overall respiratory intensity remained significantly lower than that of the other two groups, reaching a maximum of only 219.78 mg·kg^−1^·h^−1^ on day 3. As depicted in Figure 1D, soluble-protein levels in *V. volvacea* from all three experimental groups declined progressively during storage, reflecting ongoing nutrient utilisation for metabolic processes. Notably, RT experienced a rapid reduction in soluble protein, reaching 13.84 mg·g^−1^ by day 3. Contrastingly, NP maintained a higher level (15.17 mg·g^−1^) until day 4. Most significantly, CAP maintained a high soluble-protein content throughout the storage period, reaching 26.54 mg·g^−1^ even on the fourth day. These findings confirm that pre-cooling treatment effectively retards the degradation of nutritional components, thereby enhancing the shelf life of *V. volvacea*.

Figure 1E illustrates that the firmness of *V. volvacea* declined over time in all groups. The two groups stored at 15 °C and 85% relative RT exhibited a slower rate of firmness loss, whereas RT showed a rapid decline. Notably, CAP maintained a relatively high firmness during the first two days, while NP experienced a more pronounced reduction by day 2. Figure 1F shows that the browning index increased with storage duration across all groups. CAP exhibited significantly lower browning than NP, and RT displayed markedly higher browning than the other two groups stored at 15 °C and 85%. As shown in Figure 1G, the MDA content generally increased during storage, indicating progressive membrane lipid peroxidation. CAP consistently maintained the lowest MDA levels throughout the storage period, whereas RT showed a sharp increase after day 1, remaining substantially higher than the other two groups. These findings suggest that pre-cooling effectively suppresses MDA accumulation and alleviates oxidative stress, thereby reducing cellular damage to fruiting bodies. Figure 1H shows that the PPO activity increased in a fluctuating manner over time, which is consistent with the browning trend. CAP exhibited significantly lower PPO activity than NP and RT. Although RT and NP showed rapid increases in PPO activity after day 1, CAP remained at a low level until a noticeable increase on day 4—still lower than that of NP. This indicates that pre-cooling effectively inhibits PPO activation, thereby delaying enzymatic browning. Given that PPO can accelerate cell wall degradation, it may contribute to a loss of firmness. Notably, RT exhibited a sharp decline in firmness concurrent with the increase in PPO activity on day 2, whereas CAP maintained high firmness and low PPO activity for the first 3 d. The temporal correlation between increased PPO activity and decreased firmness supports a mechanistic link between enzyme activity and textural deterioration. PPO also accelerates cell wall breakdown, reducing fruit body firmness. RT exhibited a significant decrease in firmness on the second day, which coincided with the increase in PPO activity, while CAP maintained higher firmness.

### 3.2. Analysis of Energy Metabolism Indices of V. volvacea over Different Storage Periods

**Figure 2:**
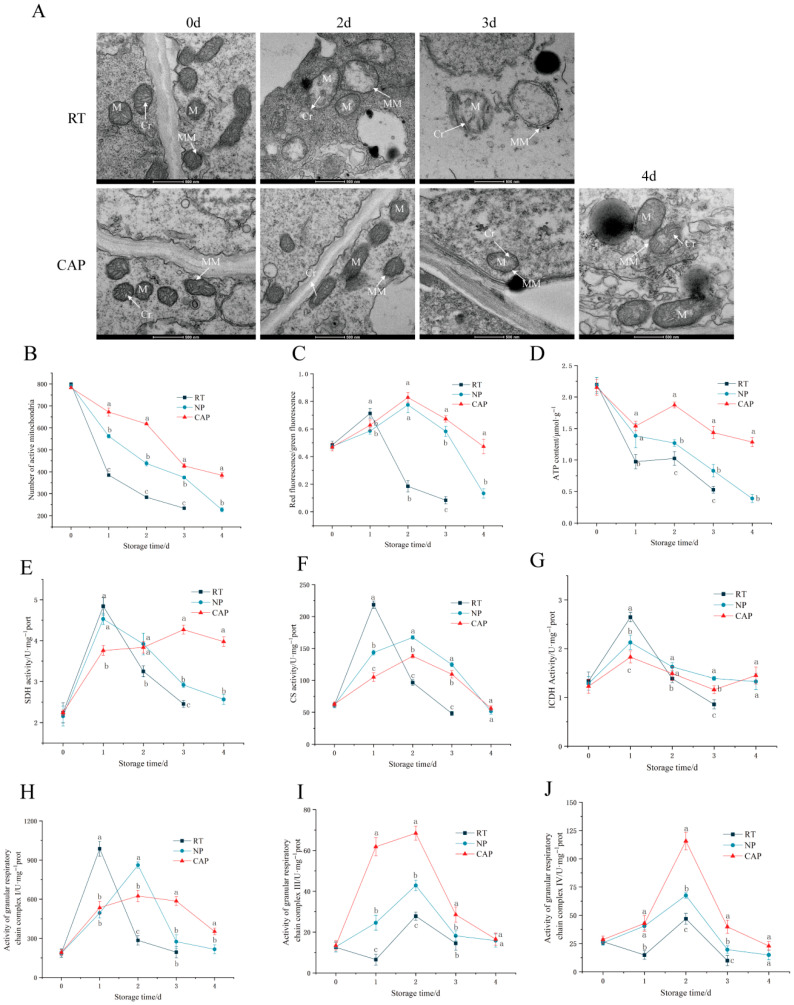
Effect of different treatments on ultrastructure (M, MM, and Cr are mitochondria, mitochondrial membrane and cristae, respectively) (**A**); number of active mitochondria (**B**); mitochondrial membrane potential (**C**); ATP content (**D**), enzyme activity of SDH (**E**), CS (**F**), and ICDH (**G**); and mitochondrial complex I (**H**), III (**I**) and IV (**J**) activity in *V. volvacea* during different periods. Different letters indicate statistically significant differeneces (*p* < 0.05).

Mitochondria serve as the principal sites for oxidative phosphorylation and ATP synthesis within cells and function as central hubs for energy metabolism and material transformation [28]. Their functional decline is closely associated with structural damage. During the postharvest quality deterioration of *V. volvacea*, the mitochondrial double membrane and inner cristae structures are altered significantly. Figure 2A illustrates that fresh *V. volvacea* exhibited intact double-membrane structures with tightly arranged inner cristae. By the second day of storage at room temperature, mitochondria exhibited marked swelling, vacuolation, and cristae fragmentation, accompanied by double-membrane disruption. By the third day, the double-membrane structure had completely disintegrated, with fragmented cristae exposed. Thus, observation of mitochondrial structure on the fourth day was deemed unnecessary. Contrastingly, the pre-cooling samples maintained intact double-membrane structures on the second day, with densely packed cristae. On the third day, the double membrane remained intact, but the cristae began to disorganise and were partially fragmented. By the fourth day, mitochondrial swelling had occurred, accompanied by double-membrane rupture in a few mitochondria and content leakage, though most mitochondrial structures remained intact.

After entering live cells, Janus Green B dye specifically accumulated on the inner mitochondrial membrane and became bound to cytochrome-c oxidase, thereby exhibiting characteristic colouration. As shown in Figure 2B, the number of active mitochondria in *V. volvacea* from all three groups decreased over storage time. In RT, a significant decrease was observed by day 1, whereas the two groups stored at 15 °C exhibited a slower decline during the early storage phase. In the later stages, CAP maintained a notably higher number of active mitochondria than NP. These findings indicate that pre-cooling treatment slows the inactivation rate of mitochondria, helping preserve their structure and function.

Mitochondria, as the core organelles for the cellular energy supply, generate membrane potential owing to the transmembrane movement of ions across biological membranes. This potential is a critical condition for maintaining oxidative phosphorylation and ATP synthesis in mitochondria and serves as a key indicator for evaluating mitochondrial functional integrity [29]. The membrane potential directly influences the mitochondrial respiratory chain electron transfer, double-membrane integrity, material transport, and structural morphology [30]. A decline in MMP is a hallmark of early apoptosis. As shown in Figure 2C, under all three treatment conditions, the MMP of *V. volvacea* exhibited an initial increase, followed by a decline over storage time. In RT, the red-to-green fluorescence ratio peaked on day 1 (0.71) and then decreased sharply. In contrast, the two groups stored at 15 °C (pre-cooling and non-pre-cooling) reached their peak ratios on day 2, with a steep decline beginning after day 3. Notably, the decrease was more gradual in CAP, indicating that pre-cooling treatment effectively delays the onset of cellular apoptosis.

ATP is the primary energy source for life activities. The ATP content in *V. volvacea* from all three groups exhibited a progressive decline during postharvest storage, reflecting a depletion of endogenous nutrients in the absence of an external supply (Figure 2D). RT displayed a rapid reduction in ATP level, reaching 0.97 µmol·g^−1^ on day 1 and maintaining lower values than the other two groups throughout the storage period. In contrast, CAP sustained relatively higher ATP concentrations, with 1.28 µmol·g^−1^ still observed on day 4—equivalent to the ATP level in NP on day 2. These findings suggest that pre-cooling treatment helps preserve energy metabolism by decreasing the rate of ATP depletion.

As shown in Figure 2E–G, compared to the NP and RT control groups, CAP treatment effectively suppressed the early surge in succinate dehydrogenase (SDH), citrate synthase (CS), and isocitrate dehydrogenase (ICDH) activities, and slowed their subsequent decline during storage. In the RT group, CS, ICDH, and SDH activities peaked sharply on day 1, followed by a rapid decline, indicating an initial metabolic burst that prematurely exhausted endogenous resources and triggered quality deterioration. In contrast, CAP treatment maintained consistently low ICDH and SDH activities throughout storage without pronounced peaks, while CS activity remained persistently lower than in the control groups. As CS and ICDH are two key rate-limiting enzymes in the TCA cycle, these results confirm that CAP suppresses TCA cycle activity. Postharvest metabolism of *V. volvacea* relies on the TCA cycle for nutrient catabolism and energy substrate supply. Maintaining the TCA cycle at an optimal metabolic rate is critical for prolonging postharvest shelf life: excessive respiratory activity during early storage accelerates the cycle, leading to rapid nutrient depletion and accumulation of metabolic byproducts, while insufficient activity fails to meet cellular energy demands, compromising tissue integrity.

Mitochondria possess a double-membrane structure, with the inner membrane hosting five complexes associated with respiration. Together with ubiquinone and cytochrome c, these five complexes form the mitochondrial respiratory chain [31]. The electron transport chain on the inner membrane participates in oxidative phosphorylation, ultimately generating H_2_O and ATP to provide the energy required for cell survival [32]. This process is the primary source of energy production within cells. Among the five complexes on the inner membrane, I, III, and IV form the main chain of the respiratory pathway, accepting electrons from various metabolic routes [33]. Figure 2H shows that the activity of the mitochondrial respiratory chain complex I in *V. volvacea* fruiting bodies from all three treatment groups exhibited a biphasic response during storage, characterised by an initial peak followed by a gradual decline. In RT, the peak activity occurred on day 1, after which it decreased rapidly. By contrast, both NP and CAP reached their maximum activities on day 2, with CAP (624.27 U·mg^−1^ prot) showing significantly lower activity than NP (861.18 U·mg^−1^ prot). Notably, CAP maintained a relatively stable activity level (534.84–624.27 U·mg^−1^ prot) between days 1 and 3, with a more gradual decline rate compared to the other two groups. As depicted in Figure 2I, the activity of mitochondrial respiratory chain complex III exhibited a marked temporal pattern across treatment groups, with the pre-cooling group demonstrating the most rapid initial increase. All groups reached their peak activities on day 2, followed by a decline phase. Between days 1 and 3, CAP maintained significantly higher activity levels than the other two groups, while RT consistently displayed the lowest activity. For complex IV, a similar temporal pattern was observed, with all groups peaking on day 2 and RT maintaining the lowest activity from day 1 onwards in Figure 2J. CAP showed no significant difference from NP on day 1 but exhibited significantly higher activity than NP from days 2 to 4. These findings indicate that pre-cooling treatment effectively modulates the activities of mitochondrial respiratory chain complexes I, III, and IV, thereby maintaining stable oxidative phosphorylation and ensuring a consistent energy supply for *V. volvacea*.

These findings demonstrate that CAP effectively regulates energy metabolism in *V. volvacea* by inhibiting the TCA cycle activity and modulating respiratory complex function, stabilizing intracellular ATP levels, and thereby extending postharvest shelf life.

### 3.3. Transcriptomic Profiling of V. volvacea Under Different Treatments During Different Storage Periods

**Figure 3:**
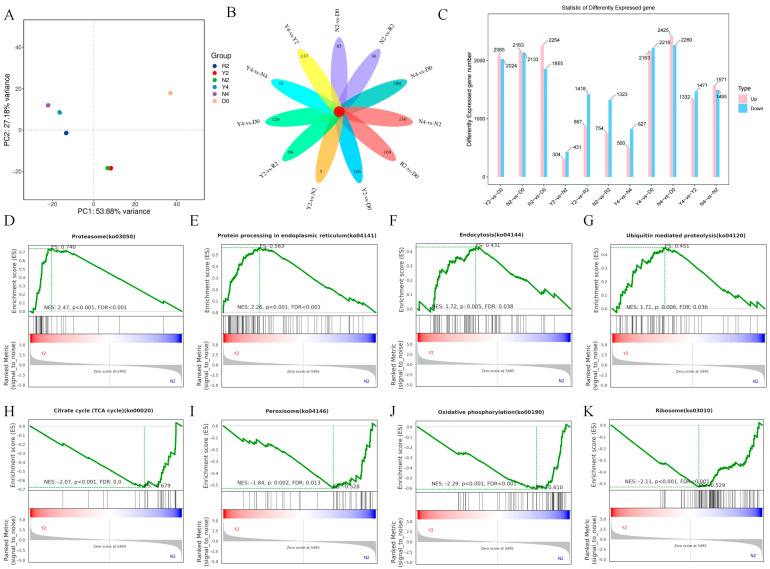
Transcriptome analysis of *V. volvacea* under different postharvest treatments. (**A**) Principal Component Analysis (PCA) of expressed genes; (**B**) Venn diagram analysis of differentially expressed genes (DEGs); (**C**) statistical summary of DEGs; (**D**) gene set enrichment analysis (GSEA) of the proteasome pathway; (**E**) GSEA of the endoplasmic reticulum protein processing pathway; (**F**) GSEA of the endocytosis pathway; (**G**) GSEA of the ubiquitin-mediated proteolysis pathway; (**H**) GSEA of the citrate cycle (TCA cycle) pathway; (**I**) GSEA of the peroxisome pathway; (**J**) GSEA of the oxidative phosphorylation pathway; (**K**) GSEA of the ribosome pathway. Abbreviations: R2, stored at room temperature for 2 d; Y2, pre-cooled and stored at 15 °C for 2 days; N2, stored at 15 °C for 2 days without pre-cooling; Y4, pre-cooled and stored at 15 °C for 4 days; N4, stored at 15 °C for 4 days without pre-cooling. D0, stored at room temperature for 0 d.

We performed transcriptome analysis on RT, NP, and CAP to obtain gene expression profiles for each group. PCA of the transcriptomic data revealed that, after 2 d of storage, RT (R2) was clearly separated from the 15 °C treatment groups (NP N2 and CAP Y2), indicating that different temperature treatments significantly altered the gene expression patterns of *V. volvacea* (Figure 3A). Under 15 °C cold stress for 2 d, N2 and Y2 showed partial overlap, suggesting that pre-cooling treatment could partially affect the cryogenic response expression pattern of *V. volvacea* (Figure 3A). After 4 d of 15 °C cold stress, N4 and Y4 were separated, indicating that prolonged cold stress could greatly influence the expression profile of *V. volvacea* (Figure 3A).

Venn analysis of DEGs showed that only 16 DEGs were shared among the different treatment groups (Figure 3B). The number of unique DEGs in Y4-vs-D0 (120) was significantly lower than that in N4-vs-D0 (189). Meanwhile, the number of unique DEGs in Y2-vs-N2 was the lowest, with only five genes. These results further suggest that different treatment methods alter the cold response mode of *V. volvacea*. Statistical analysis of the number of DEGs showed that the number of downregulated genes in Y4-vs.-N4 was 827, significantly higher than that of the upregulated genes (500). Similar results were observed in Y2-vs.-N2 and Y2-vs.-R2. These results indicate that pre-cooling treatment has an inhibitory effect on the gene expression of *V. volvacea* under cold stress (Figure 3C).

To further investigate the molecular mechanism by which pre-cooling treatment improves the cold stress tolerance of *V. volvacea*, we conducted GSEA of expressed genes for a comparative analysis of representative treatments. GSEA of Y2-vs.-N2 showed that the pre-cooling treatment enhanced the activity of pathways such as proteasomes (Figure 3D), protein processing in the endoplasmic reticulum (Figure 3E), endocytosis (Figure 3F), and ubiquitin-mediated proteolysis (Figure 3G), suggesting that cells initiate multiple stress adaptation mechanisms to maintain protein homeostasis, supporting efficient energy reserve utilization. GSEA of Y2-vs.-N2 also revealed that pre-cooling treatment reduced the activity of pathways including the citrate cycle (Figure 3H), peroxisome (Figure 3I), oxidative phosphorylation (Figure 3J), and ribosome (Figure 3K), indicating a strategic reduction in cellular energy consumption to enhance postharvest resilience. In addition, GSEA of R2-vs.-D0 showed that the activity of pathways such as the citrate cycle, oxidative phosphorylation, and ribosome was upregulated during room-temperature storage (Figure S1). Similarly, GSEA of N2-vs.-D0 also showed the upregulation of these pathways under room-temperature storage (Figure S2). Combined with the optimal phenotype of Y2 (Figure 1A), these results suggest that pre-cooling treatment adapts to cold environments by decreasing the TCA activity. GSEA of Y4-vs.-Y2 showed that the pre-cooling treatment upregulated the activity of pathways such as the citrate cycle and oxidative phosphorylation, while inhibiting the activity of pathways such as protein processing in the endoplasmic reticulum under 4 d of cold stress (Figure S3). These findings indicate that prolonged cold stress impairs normal protein turnover mechanisms, even as cells exhibit elevated energy consumption to sustain stress responses. These results suggest that pre-cooling treatment can, to a certain extent, help *V. volvacea* maintain energy metabolism homeostasis at low temperatures.

### 3.4. Validation Results of qPCR Assays

**Figure 4:**
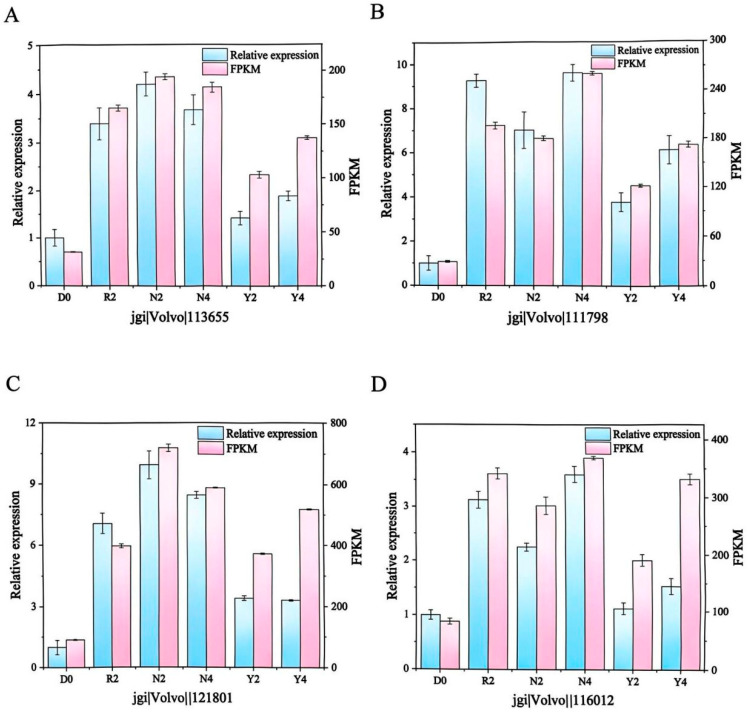
qPCR verification results of differentially expressed genes. (**A**–**D**) respectively show the expression levels and FPKM values of citrate synthase (jgi|Volvo1|113655), isocitrate dehydrogenase (jgi|Volvo1|111798), aconitate hydratase (jgi|Volvo1|121801), and mitochondrial respiratory chain complex IV (jgi|Volvo1|116012). Definitions of abbreviations for different treatments are provided in the legend of Figure 3.

Building on our previous transcriptomic analysis showing that pre-cooling treatment maintains energy metabolism, we selected four energy metabolism-related DEGs involved in the TCA cycle and oxidative phosphorylation pathways for qPCR validation, aiming to confirm the reproducibility and accuracy of our transcriptomic data. These genes include CS (jgi|Volvo1|113655), ICDH (jgi|Volvo1|111798), aconitase hydratase (jgi|Volvo1|121801), and the mitochondrial respiratory chain complex IV (jgi|Volvo1|116012). FPKM (Fragments per Kilobase of transcript per Million mapped reads) is a widely used normalization metric in transcriptomics that adjusts raw fragment counts for both gene length and sequencing depth, providing length- and library-size–normalized estimates of transcript abundance [34]. RNA-seq analysis revealed that the FPKM expression trends of these four genes aligned with those observed using qPCR (Figure 4). Pearson correlation analysis further revealed a strong positive correlation in expression fold changes between the two platforms (Figure S4), validating the reliability of our transcriptomic sequencing data. Compared with the other treatments, CAP significantly downregulated the expression of all four genes at 2 d of storage, indicating the inhibition of energy metabolism. Notably, the expression of these genes was upregulated in the CAP-treated group at the 4 d storage stage. This distinct transcriptional pattern suggests that CAP initially decelerates TCA cycle kinetics, thereby conserving endogenous nutrient reserves to support sustained energy production during subsequent storage phases.

Furthermore, pre-cooling exerted a protective effect on the integrity of the inner mitochondrial membrane. It delayed the substantial upregulation of the alternative oxidase gene (jgi|Volvo1|111228), potentially mitigating oxidative stress-induced cellular damage. Additionally, the expression-change rate of dihydroorotate dehydrogenase (DHODH, jgi|Volvo1|117577) was significantly lower in the pre-cooling group, indicating a deceleration in cell division. Crucially, pre-cooling ensured sustained upregulation of genes associated with mitochondrial respiratory chain complexes throughout the storage period. This coordinated transcriptional regulation maintained efficient electron transport and oxidative phosphorylation, thereby ensuring a continuous cellular energy supply. Collectively, these molecular adaptations underpin the efficacy of pre-cooling in prolonging the postharvest shelf life of *V. volvacea* by optimising energy metabolism and preserving mitochondrial function.

**Figure 5:**
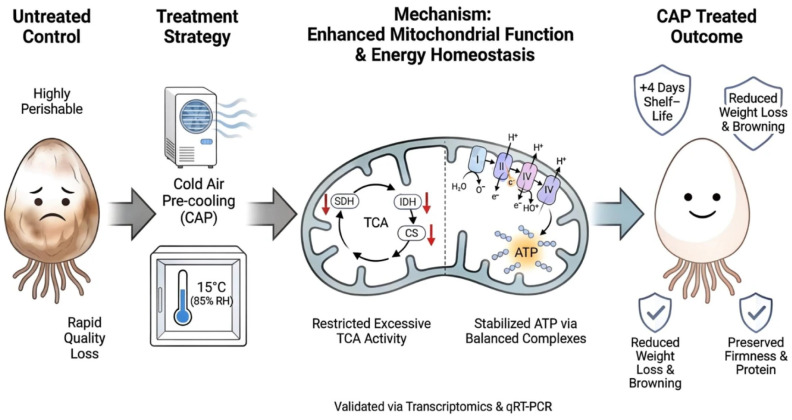
Schematic illustration of the mitochondrial mechanisms underlying CAP-mediated quality preservation in *V. volvacea*. CAP treatment enhances mitochondrial function and maintains energy metabolism homeostasis to delay deterioration. This is achieved primarily by restricting excessive TCA cycle activity via the downregulation of key enzymes (SDH, IDH, and CS) and concurrently stabilizing cellular ATP levels by balancing respiratory complex activities. Red arrows indicate down-regulated expression.

Postharvest quality deterioration in fruits and vegetables is closely linked to energy metabolism dysregulation, as evidenced by multiple horticultural studies [35]. For *V. volvacea*, maintaining a sufficient energy supply is critical for preserving postharvest quality and delaying senescence, with energy metabolism identified as a core regulatory pathway that extends shelf life [36,37,38]. This aligns with findings in bananas and *Lyophyllum decastes*, where cellular energy homeostasis is essential for enhancing chilling tolerance [39,40]. Specifically, prior research on *V. volvacea* confirmed that cold stress disrupts mitochondrial structure and function, leading to intracellular energy deficits and accelerated postharvest decay [36]. Our results demonstrate that CAP can maintain mitochondrial function by enhancing mitochondrial counts, membrane potential, and ATP content (Figure 2D). The diagram illustrates that CAP treatment maintains quality by enhancing mitochondrial function (Figure 5). Specifically, CAP regulates energy metabolism through two synergistic pathways: (1) restricting excessive tricarboxylic acid (TCA) cycle activity by downregulating key enzymes, including SDH, IDH, and CS; and (2) stabilizing ATP levels by balancing the activities of respiratory complexes. These mechanisms maintain energy metabolism homeostasis, ultimately extending shelf life and reducing postharvest deterioration.

However, the sequential application of pre-cooling followed by precise environmental control remains relatively cumbersome for practical implementation. Therefore, future research should focus on developing more streamlined, integrated preservation technologies or simplified protocols to improve commercial feasibility. Although the present study identified key genes and metabolic pathways associated with postharvest senescence via transcriptomic analysis, it primarily established correlative relationships. To translate these findings into targeted preservation strategies, subsequent studies should prioritise functional validation. Specifically, the direct modulation of enzymatic activities within the TCA cycle and oxidative phosphorylation pathway—based on the candidate genes identified herein—warrants further investigation. Targeted inhibition or stabilisation of these critical metabolic nodes may effectively decelerate energy depletion and senescence, thereby extending the shelf life of *V. volvacea*. Finally, integrating physiological interventions such as pre-cooling with molecular approaches targeting core energy metabolism represents a promising frontier for advanced postharvest preservation of this highly perishable commodity.

Although this study provides a robust mechanistic framework for the preservation of *V. volvacea* at the physical and transcriptomic levels, we acknowledge certain limitations that open avenues for future investigation. Firstly, while we focused on structural integrity, the characteristic rapid loss of umami and development of off-flavors (such as ethanol or acetaldehyde) during postharvest storage are critical quality determinants. High-impact studies in this field increasingly rely on volatile compound profiling to track flavor degradation. Future work should therefore incorporate sensory evaluation and gas chromatography–mass spectrometry (GC-MS) analysis to correlate the observed preservation of texture with the retention of aroma profiles. Secondly, given the unique autolysis mechanism of *V. volvacea*—which involves liquefaction driven by cell wall-degrading enzymes like chitinases and β-glucanases—our gene expression data provides only a transcriptional perspective. Subsequent studies are needed to directly measure the enzymatic activities of these hydrolases and to correlate energy levels with the suppression of chitinase expression. Such data would significantly strengthen the model of structural stability proposed here.

Finally, our current analysis relies heavily on transcriptomics to infer metabolic changes. To validate these findings and confirm that gene downregulation indeed results in reduced metabolic flux, future investigations employing targeted metabolomics (e.g., quantifying intermediates such as citrate and malate) will be essential. Integrating these multi-omics datasets would provide a more comprehensive picture of the preservation mechanism.

Despite these considerations, the present work successfully establishes a critical foundation, offering high-quality mechanistic insights that can guide future multi-disciplinary research on *V. volvacea* preservation.

## 4. Conclusions

This study demonstrates that CAP treatment (15 °C, 85% RH) significantly extends the shelf life of *V. volvacea* by 4 days. CAP effectively preserves postharvest quality by reducing browning and weight loss, maintaining firmness, and inhibiting MDA accumulation and PPO activity. Mechanistically, CAP maintains energy homeostasis by enhancing mitochondrial abundance and membrane potential. While it downregulates TCA cycle enzymes (SDH, IDH, and CS) and modulates respiratory complexes (I, III, and IV), CAP ensures stable ATP levels, thereby delaying the senescence of *V. volvacea*.

## Figures and Tables

**Table 1:** Primers used in qPCR.

Gene ID	Forward Primer (5′ to 3′)	Reverse Primer (5′ to 3′)
GAPDH	GGTTCCCATCCTTTCCAGC	TCGCCTTGAGAAGCCTACAG
jgi|Volvo1|118124	GTCCCAGGCGGTCCCATCTAC	GGTAGGAGCCATGTATGCGTGAAG
jgi|Volvo1|116012	CACTGCTGCTCGTCCCACAAC	TAGCGTTCGGAGAAAGCCTCAAAC
jgi|Volvo1|121801	CGACAGCGACTGCGAAGTATGG	CATACCAAGACCGCCAGCATTAGG
jgi|Volvo1|113655	CCGCCCTCTCTGATCCCTTCC	CTGAGCCACACGAGCACTTCTTG
jgi|Volvo1|111798	TTGAGCCAGGATGTCGTCATGTTG	GAACGTCGCCGCAGCTATATCG

## Data Availability

The original contributions presented in the study are included in the article/Supplementary Material, further inquiries can be directed to the corresponding authors.

## Supplementary Materials

The following supporting information can be downloaded at: https://www.mdpi.com/article/10.3390/foods15061077/s1, Figure S1: GSEA of R2-vs-D0; Figure S2: GSEA of N2-vs-D0; Figure S3: Y4-vs-Y2; Figure S4: Pearson correlation coefficients between qPCR relative expression levels and transcriptome FPKM values of the validated DEGs.
