# Supplementary material for: Cold Air Pre-Cooling Extends Postharvest Shelf Life of Volvariella volvacea by Maintaining Energy Metabolism Homeostasis
Source: Foods. 2026 Mar 19;15(6):1077. doi: 10.3390/foods15061077 (PMC13025600; doi:10.3390/foods15061077)
Supplement: Supplementary file 1 [file foods-15-01077-s001.zip › foods-4194770-supplementary.pdf]

Supplementary Figure

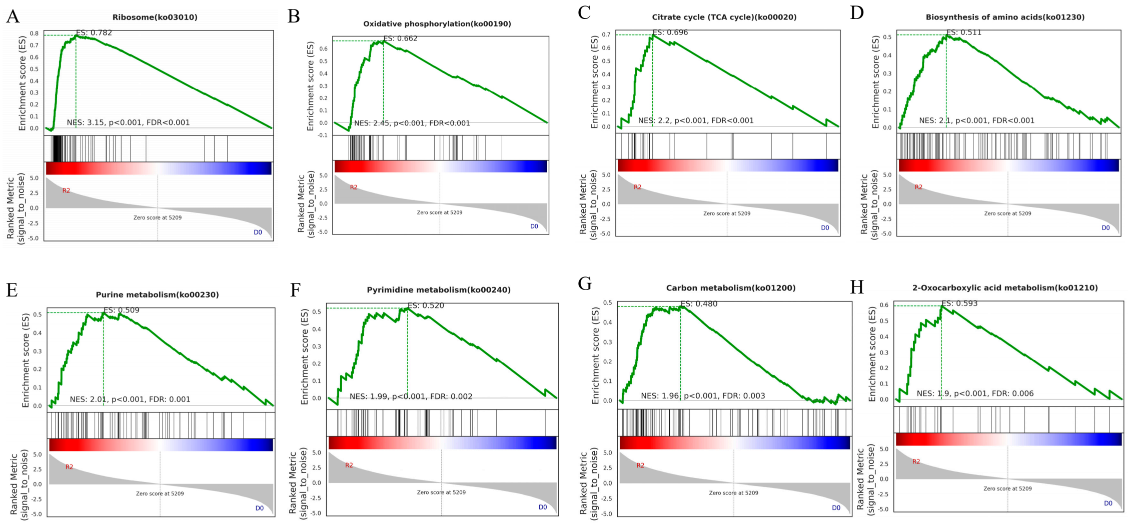

Figure S1. GSEA of R2-vs-D0. Abbreviations: R2, stored at room temperature for 2 d; D0, harvested at 0 day (D0).

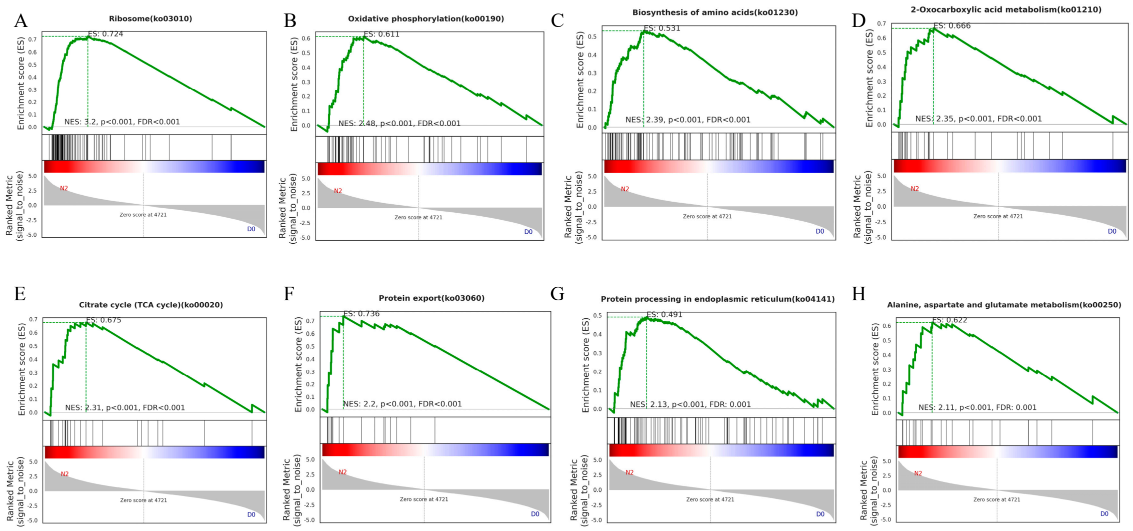

Figure S2. GSEA of N2-vs-D0. N2, stored at 15°C for 2 d without precooling treatment; D0, harvested at 0 day.

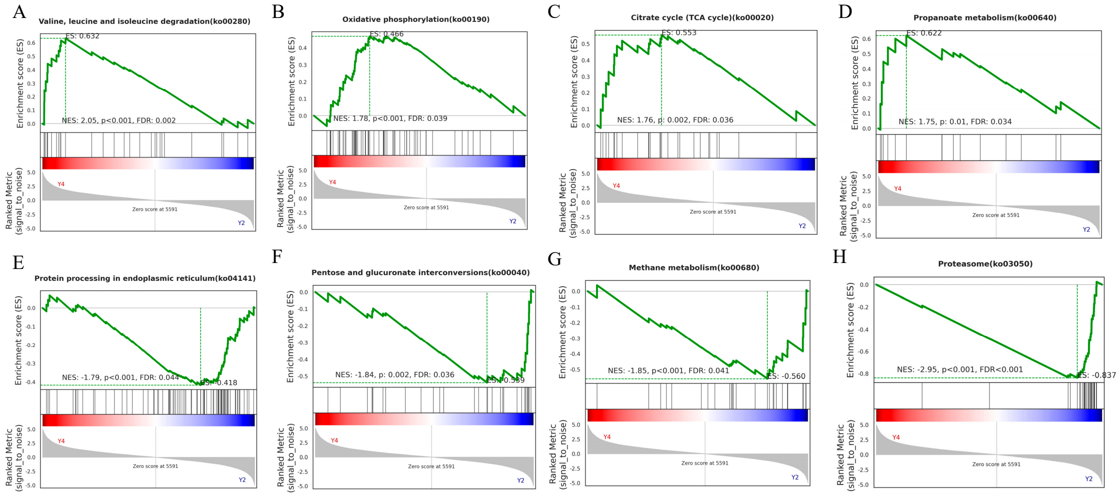

Figure S3. GSEA of Y4-vs-Y2. Y2, precooled and stored at 15° C for 2 d; Y4, precooled and stored at 15° C for 4 d.

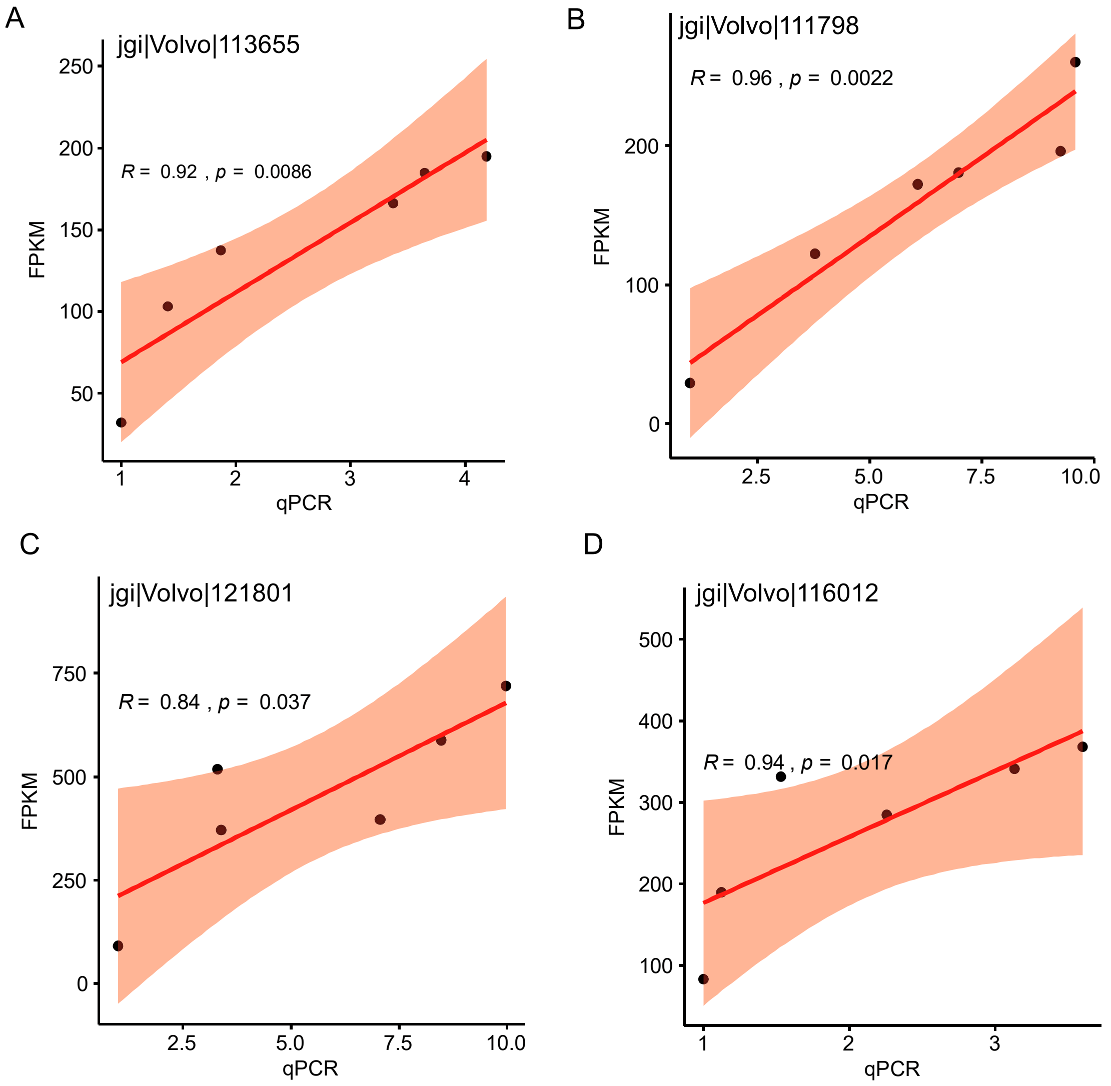

Figure S4. Pearson correlation coefficients between qPCR relative expression levels and transcriptome FPKM values of the validated DEGs. Figures (A)-(D) respectively show the expression levels and FPKM values of citrate synthase (jgi|Volvo1|113655), isocitrate dehydrogenase (jgi|Volvo1|111798), aconitate hydratase (jgi|Volvo1|121801), and mitochondrial respiratory chain complexⅣ (jgi|Volvo1|116012).
